# A cluster randomized controlled trial to evaluate the effectiveness of the clinically integrated RHL evidence -based medicine course

**DOI:** 10.1186/1742-4755-7-8

**Published:** 2010-05-14

**Authors:** Regina Kulier, Khalid S Khan, A Metin Gulmezoglu, Guillermo Carroli, Jose G Cecatti, Maria J Germar, Pisake Lumbiganon, Suneeta Mittal, Robert Pattinson, Jean-Jose Wolomby-Molondo, Anne-Marie Bergh, Win May

**Affiliations:** 1 WHO Collaborating Centre in Research Synthesis, Birmingham University, Birmingham, UK; 2 Director, WHO Collaborating Centre in Research Synthesis, Birmingham University, Birmingham, UK; 3 UNDP/UNFPA/WHO/World Bank Special Programme of Research, Development and Research Training in Human Reproduction, Department of Reproductive Health and Research, World Health Organization, Geneva, Switzerland; 4 CREP - Centro Rosarino de Estudios Perinatales, Rosario, Argentina; 5 UNICAMP - Department of Obstetrics and Gynecology from the University of Campinas, Brazil; 6 Department of Obstetrics and Gynaecology, University of the Philippines, Manila, Philippines; 7 Department of Obstetrics and Gynaecology, Khon Kaen University, Khon Kaen, Thailand; 8 Department of Obstetrics and Gynaecology, All India Institute of Medical Sciences, New Delhi, India; 9 Department of Obstetrics and Gynaecology, University of Pretoria, Pretoria, South Africa; 10 Department of Obstetrics and Gynaecology, University Hospital of Kinshasa, Democratic Republic of Congo; 11 University of South California, USA

## Abstract

**Background and objectives:**

Evidence-based health care requires clinicians to engage with use of evidence in decision-making at the workplace. A learner-centred, problem-based course that integrates e-learning in the clinical setting has been developed for application in obstetrics and gynaecology units. The course content uses the WHO reproductive health library (RHL) as the resource for systematic reviews. This project aims to evaluate a clinically integrated teaching programme for incorporation of evidence provided through the WHO RHL. The hypothesis is that the RHL-EBM (clinically integrated e-learning) course will improve participants' knowledge, skills and attitudes, as well as institutional practice and educational environment, as compared to the use of standard postgraduate educational resources for EBM teaching that are not clinically integrated.

**Methods:**

The study will be a multicentre, cluster randomized controlled trial, carried out in seven countries (Argentina, Brazil, Democratic Republic of Congo, India, Philippines, South Africa, Thailand), involving 50-60 obstetrics and gynaecology teaching units. The trial will be carried out on postgraduate trainees in the first two years of their training. In the intervention group, trainees will receive the RHL-EBM course. The course consists of five modules, each comprising self-directed e-learning components and clinically related activities, assignments and assessments, coordinated between the facilitator and the postgraduate trainee. The course will take about 12 weeks, with assessments taking place pre-course and 4 weeks post-course. In the control group, trainees will receive electronic, self-directed EBM-teaching materials. All data collection will be online.

The primary outcome measures are gain in EBM knowledge, change in attitudes towards EBM and competencies in EBM measured by multiple choice questions (MCQs) and a skills-assessing questionniare administered eletronically. These questions have been developed by using questions from validated questionnaires and adapting them to the current course. Secondary outcome measure will be educational environment towards EBM which will be assessed by a specifically developed questionnaire.

**Expected outcomes:**

The trial will determine whether the RHL EBM (clinically integrated e-leraning) course will increase knowledge, skills and attitudes towards EBM and improve the educational environment as compared to standard teaching that is not clinically integrated. If effective, the RHL-EBM course can be implemented in teaching institutions worldwide in both, low-and middle income countries as well as industrialized settings. The results will have a broader impact than just EBM training because if the approach is successful then the same educational strategy can be used to target other priority clinical and methodological areas.

**Trial Registration:**

ACTRN12609000198224

## Background

Research is constantly increasing medical knowledge. For this increased knowledge to be useful in improving the health of populations it should be added to the existing knowledge pool, critically evaluated for validity and implemented by health care professionals. In recent years it is increasingly recognized that improvements in knowledge and skills of health care personnel and improvements in health care outcomes is best accomplished by employing evidence-based medicine (EBM) as part of everyday work. EBM promotes health care decisions based on the current best (valid and relevant) evidence. To achieve this, EBM curricula need to inculcate amongst learners the skills to gain, assess, apply, integrate and communicate new scientific knowledge into clinical decision-making. There is much debate about the effectiveness of various EBM teaching and learning methods and outcomes. Computer-based learning has been shown to be as effective as face-to face, lecture based sessions in improving knowledge [1,2]. There is a lack of consensus, however, as to what methods constitute the best educational practice: a practice that results not only in improvement of basic educational outcomes, such as knowledge and appraisal skills but also in attitudes and behaviour, which, ultimately leads to improved practice [3,4].

Empirical and theoretical evidence suggests that there is a hierarchy of teaching and learning activities in terms of their educational effectiveness: Level 1, interactive and clinically integrated activities; Level 2(a), interactive but classroom based activities; Level 2(b), didactic but clinically integrated activities; and Level 3, didactic, classroom or standalone teaching [3]. Workshop-based teaching, probably one of the most common forms of postgraduate teaching, can at best achieve level 2. Workshops can be interactive but it is the provision of clinically integrated activities that seem to have more sustained effects and have advantages over workshop-based programmes. Such programmes are few and variable in their quality.

A clinically integrated EBM course for teaching postgraduates was developed by the European Union-EBM Unity Project, a consortium of eleven European partners within the framework of Leonardo da Vinci vocational programme of the European Union http://ebm-unity.pc.unicatt.it/[5]. This course is learner-centred, problem-based and has integrated e-learning with clinical activities in the workplace. When successfully implemented, the course is designed to provide just-in-time learning through on-the-job-training, with the potential for teaching and learning to directly impact on practice and workplace environment. The course was formally piloted for feasibility in five European countries, in different medical specialties and different languages and tested in a cluster randomized controlled trial in two countries with promising results [6,7]. However, the generic nature of this course makes application in specific clinical specialties tedious. There is a need to develop specialty- specific courses and to further evaluate this approach rigorously in appropriate settings.

The World Health Organization has published the *WHO Reproductive Health Library *(RHL), an evidence-based specialist library in sexual and reproductive health since 1997. RHL is disseminated extensively in low and middle-income countries to approximately 15,000 users every year. RHL contents are Cochrane systematic reviews in high priority topics mainly in maternal and perinatal health and fertility regulation complemented by commentaries, practical guidance documents and educational videos on techniques to implement effective practices.

In addition, a course on *Evidence-based Decision-making in Reproductive Health *including RHL content has been developed in past years as part of capacity strengthening efforts. This course includes six powerpoint presentations on evidence-based decision-making and a manual with the presentations. Several workshops have been conducted using these materials in the past seven years.

The EU-EBM Unity Project materials were revised to adapt into a sexual and reproductive health context and include RHL as a resource. The revision has made this innovative approach to postgraduate teaching suitable for implementation at obstetrics and gynaecology departments in low and middle-income countries that collaborate with HRP/RHR. As mentioned above, the promising nature of the approach and the relatively weak evidence base to recommend it widely is the principal reason behind the development of this protocol to conduct a RCT to evaluate its effects on various relevant outcomes.

Educational activities within a clinical environment primarily aim to improve the knowledge, attitudes and skills of clinical staff that will lead to improved health care practices and health outcomes of the patients. Given the multitude of educational activities that come in various formats (i.e. didactic, interactive, face-to-face, electronic (1)) it should be an implicit aim of these activities that they result in creating an environment that is more conducive to in-service learning in addition to their more immediate objectives. The context in which these educational activities and learning by staff and students take place is defined as the 'educational environment'. A single educational project may not cause major changes in the educational environment and many other factors related to health system functioning and cultural characteristics may play a role in making the environment more educationally-friendly or not. Nevertheless, measuring the educational environment is important both as an end-point as well as an explanatory factor for evaluating educational interventions.

### Rationale for this trial

Evidence-based reproductive health care requires clinicians to engage with use of evidence in decision-making. To genuinely get involved, healthcare professionals need to effectively incorporate contemporaneous research-based information in the clinical setting. The proposed research project represents part of a continued programme of activities at HRP/RHR aimed at improving the quality of care and capacity-strengthening for evidence-based decision-making in low and middle-income countries. Such research is timely and important because health care workers in many under-resourced settings face difficulties in providing good quality care, keeping their knowledge up-to-date and having the skills to interpret and implement this knowledge. If effective, the clinically integrated e-learning approach can be scaled up without major investment at teaching institutions worldwide. The studies available to date have been exclusively conducted in developed countries. Before engaging in large scale implementation the benefits of this integrated approach should be demonstrated through rigorous research in relevant settings. The hypothesis is that the proposed RHL-EBM integrated e-learning course (experimental group) will improve participants' knowledge, skills and attitude, as well as institutional practice and educational environment, as compared to the use of standard postgraduate educational resources for EBM teaching.

### Previous similar studies

We identified a systematic review including 23 studies comparing standalone with clinically integrated teaching in postgraduate teaching in EBM [8]. Most studies reported on knowledge, fewer on skills, (i.e. critical appraisal skills) attitudes and behaviour towards EBM. None of the studies evaluated health outcomes. Results showed that both standalone and clinically integrated courses can improve knowledge. Clinically integrated courses also improved skills, attitudes and behaviour towards EBM. However, the studies included used different teaching methods and assessment tools, and no study compared directly standalone with integrated approach. While the evidence of the review looks promising, more robust evaluation is needed comparing standalone with clinically integrated teaching.

A recent systematic review assessed the effectiveness of EBM teaching on knowledge, skills and attitude/behaviour regarding EBM [9] in postgraduates. The review included 24 studies of different designs, all studies were conducted in developed countries, and most had small sample size and provided little detail about assessment tools and actual intervention. These weaknesses point towards a need for appropriate sized randomized controlled trials and trans-culturally adapted measurement instruments.

### Objectives

The main objective is to evaluate whether the RHL-EBM course is effective in improving knowledge, skills and competencies as compared to passive dissemination of resource materials. A secondary objective is to validate an EBM educational environment tool.

## Methods

### General

A cluster randomized design is proposed. Such a design is more appropriate for this intervention since the intervention will be implemented at the teaching unit level and the outcome assessments will be measured similarly at the institutional level. In addition, individual randomization can invalidate such an intervention due to contamination [10], whereby control participants have access to and use experimental intervention to enhance their learning if they are working in the same environment.

### Participants

Participants are training institutions in obstetrics and gynaecology in the participating countries. Hospitals belonging to the same training institution will form the cluster. Principal investigators from each country will determine the number of potentially eligible training units/clusters in their countries by contacting the head of the specialist training unit and the local authorities (if required). A baseline survey of staffing levels and current postgraduate teaching programmes has been conducted to determine the number of units that can meet eligibility criteria.

### Inclusion criteria

Training units that can provide at least four junior residents/registrars (postgraduate trainees) who have not yet been exposed to structured, formal EBM training and who will be available for the duration of the trial will be eligible. Brief descriptive characteristics of each participating junior postgraduate trainee will be collected.

Each institution needs to identify a 'facilitator' who will be able to work with the participants during the trial period. The facilitator will be someone working in the same department with the participants but will be a senior staff member preferably a specialist in obstetrics and gynaecology who is knowledgeable about basic EBM principles.

### Recruitment and allocation

The training institutions (clusters) identified by the principal invstigators and fulfilling the inclusion criteria will be asked to participate in the trial. The random allocation scheme will be generated at HRP/RHR using computer-generated random numbers. Each cluster will be allocated to either of two groups: RHL-EBM course (intervention 1) or passive dissemination of EBM teaching materials (intervention 2). Due to the nature of the intervention and design it will not be possible to blind the research teams or the participants of their allocated group. However, the participants will only be informed about an educational programme evaluation and will not be told which groups are compared. The individual trainees will receive similar information on a written information sheet.

### Interventions

The project aims to evaluate the effects of the experimental intervention (RHL-EBM clinically integrated e-learning course) over passive dissemination of resources that have very similar content and the same learning objectives. It is acknowledged that other EBM teaching activities can also take place independently at the participating institutions. Information on those activities will be recorded systematically. The contents and presentation of both intervention courses were refined and finalized at two meetings of the steering committee that includes experts in research methodology, medical education and obstetrics and gynaecology.

### Intervention 1: RHL-EBM Course

The RHL-EBM clinically integrated e-learning course will be the experimental intervention. The course content has been developed following well established guidelines when planning a curriculum [11]. The Course is described in detail in Tables 1 and 2. Briefly, the teaching takes place in the clinical environment and the postgraduate trainee obtains the theoretical knowledge from the interactive e-learning materials, completes assignments and interacts with her/his facilitator throughout the process.

**Table 1:** Intervention 1: WHO RHL EBM clinically integrated e-learning course

Description of the intervention	A course developed in a collaborative project involving 8 international partners. This course is based on the *Leonardo da Vinci - Community Vocational Training Action Programme *initiated and supported by the European Commission. http://ebm-unity.pc.unicatt.it.
**Aim**	To familiarise course participants with evidence based medicine (EBM) basics to help incorporate evidence from systematic reviews into practice.

**Target participants**	Trainee doctors.

**Learning objectives**	Upon the completion of the course, participants should be competently able to: •generate structured questions arising from clinical problems in practice •search relevant literature, identifying systematic reviews wherever possible •assess the quality (validity) of systematic reviews and primary research included within them •assess the applicability of research findings in clinical practice •identify possible barriers when implementing the output from above activities into clinical practice and apply strategies to overcome them.

**Reading/Learning Resource**	A study guide outlining the course and providing learning exercises/assignments and E-learning sessions. 5 modules provide learning materials for undertaking the exercises in the study guide: Module 1: Asking clinical questions Module 2: Searching the evidence Module 3: Critical appraisal of systematic reviews Module 4: Applicability of the evidence to the patient Module 5: Implementation of evidence into practice The WHO Reproductive Health Library CD-ROM/internet version

**Learning/teaching methods**	Participant initiated (supported by a facilitator) teaching and learning in a clinical setting Clinical facilitator will guide participants in a clinical setting: •Identifying learning opportunities in a clinical setting •Directing appropriate use of learning resources •Providing feedback on learning exercises/assignments Participants will pursue independent study using the study guide and e-learning modules directed/facilitated by the facilitator and will undertake summative assessments.

**Student Directed Learning**	2 - 3 hours - e-learning.

**Contact time**	20 hours - total including assignments, feedback, and assessments.

**Assessments**	Feedback on assignments Multiple choice questions to test knowledge Questionnaire to test attitudes OSCE to test competencies Questionnaire on educational environment (EBMEEM).

**Table 2:** Details about the activities (intervention)

1. E-learning sessions	The web-based e-learning tools are structured as short sessions (one or more per module) that each can be completed in 15-25 minutes by the participant. These sessions are aimed to give a basic understanding about the concepts involved in each module. Multiple choice questions for self-assessment will be incorporated in the e-sessions.
**2. Learning/teaching in a clinical setting**	This part is guided by a facilitator during the wardround and includes identification of learning opportunities in a clinical setting. Participants will, by using practical examples, apply the concepts of evidence-based medicine in a real clinical case scenario and be able to discuss with the facilitator and their colleagues.

**3. Formative assessment (assignments, OSCE)**	The participant needs to complete formative assignments which consist of MCQs, assignments for each module and questionnaires (attitude, educational environment) and OSCEs at the end of the course. After having studied each module, completed assignments need to be sent to the tutor who will provide feedback, which will serve as discussion background between participant and facilitator. The facilitator will decide when the assignment has been completed successfully. OSCEs will need to be completed at the end of the course.

**4. Summative assessment**	Participants are assessed at the end the course by multiple choice questions (MCQs) which have to be completed pre-course and four weeks after the end of the course. A questionnaire to assess participants' attitudes towards EBM and a questionnaire to assess the educational environment will be taken pre-course (at baseline) and at the end of the course. This will not count towards the participant's overall assessment. Participants will be provided with a password to gain access to the online MCQs. For this, a suitable place at the learner's department or institute providing internet access should be identified. The test has to be taken in presence of the tutor or a person designated by the tutor and the use of additional materials is not allowed. Once the pre-course MCQs have been completed, the partcipant will have access to the online e-sessions and related materials. Assignments need to be completed for each module before moving on to the next module. Assessment (MCQs for all five modules)) will need to be completed at the beginning of the course and at the end.

Descriptive information about facilitators' position in the unit, professional qualification and possible EBM teaching activities will be collected. There should be one facilitator per group of 5 to 10 postgraduate trainees. At each training institution one facilitator will be responsible for trial related activities. Facilitators will interact with the participants providing feedback on their assignments, and facilitate discussions during ward rounds/discussions and journal clubs. Facilitators will receive a handbook that will help them to guide the participant.

### Intervention 2: Self-directed learning (Passive dissemination of EBM teaching materials)

The RHL-EBM Course will be compared to passively disseminated EBM resources from the WHO RHL workshop-based course that has the same learning objectives. The materials include 6 PowerPoint presentations. The content of the presentations are similar to the content of the RHL-EBM Course. It is detailed described in Table 3. The PowerPoint presentations focus on:

**Table 3:** Intervention 2: Self-directed learning (Passive dissemination of RHL-EBM documents)

Description of the intervention	A course developed by WHO RHR in collaboration with South African Cochrane Centre. This course is implemented either through workshops or simple dissemination of course materials.
**Aim**	To familiarise course participants with evidence based medicine (EBM) basics to help incorporate evidence from systematic reviews into practice.

**Target participants**	Trainee doctors.

**Learning objectives**	Upon the completion of the course, participants should be competently able to: •generate structured questions arising from clinical problems in practice •search relevant literature, identifying systematic reviews wherever possible •assess the quality (validity) of systematic reviews and primary research included within them •assess the applicability of research findings in clinical practice

**Reading/Learning Resource**	6 Powerpoint presentations WHO Reproductive Health Library CD-ROM/internet version

**Learning/teaching methods**	Participant initiated learning using electronic resources provided

**Student Directed Learning**	e-learning (approximately 10 hours learning time)

**Contact time**	No structured planned contact time. Participants will have access to the online powerpoint presentations for 8 weeks after they have completed the baseline assessment.

**Assessments**	Multiple choice questions to test knowledge Questionnaire to test attitudes OSCE to test competencies Questionnaire on educational environment (EBMEEM).

• formulating clinical answerable questions;

• searching effectively for evidence using tools such as the Reproductive Health Library and the Cochrane Library;

• critically appraise clinical evidence for its validity and applicability;

• understanding basic effect measures such as Relative Risk and Numbers Needed to Treat.

The basic differences between experimental (intervention 1) and control (intervention 2) groups are outlined in Table 4.

**Table 4:** Intervention and control group

	Experimental group	Control group
**Before**	Introduction and consent MCQs M1-M5 Attitude questionnaire Educational environment measurement tool	Introduction and consent MCQs M1-M5 Attitude questionnaire Educational environment measurement tool

**Implement interventions**	•RHL EBM course over maximum 8 weeks (5 modules, completion of assignments for each module/week) •Current local teaching	•RHL and workshop-based course materials handed out to each participant •Current local teaching

**Assessment**	•MCQs: 3-4 weeks after the end of the course •Attitudes: questionnaire 3-4 weeks after the end of the course •Competencies: OSCE 4 weeks after the end of the course •Educational environment: 4 weeks after the end of the course	•MCQs: 12 weeks after baseline assessment •Attitudes: 12 weeks after baseline assessment •Competencies: OSCE 12 weeks after baseline assessment •Educational environment: 12 weeks after baseline assessment

### Outcome measures

The primary outcome measures are:

**Gain in EBM knowledge **measured by multiple choice questions (MCQs) that have been shown to have face/content validity in relation to course objectives and discrimination capacity in previous studies. Multiple choice questions have been developed by using questions from validated questionnaires and adapting them to the current course [4,12,13].

**Change in attitudes **towards EBM and competencies in EBM. Attitudes towards EBM will be measured by a validated questionnaire. Previously validated questions to assess attitudes towards EBM will be used [4].

**Skills competence **in applying EBM principles will be measured by *Objective Structured Clinical Examination *(OSCE). The OSCE is a well established way to assess clinical competency. In this case, EBM-skills related questions following the OSCE structure will be developed for completion electronically.

As a secondary outcome measure, we will assess the educational environment towards EBM by using a questionnaire (EBMEEM). The EBMEEM development is based on previously validated environment measurement tools in different clinical settings [14,15]. The trial will also serve to validate the tool in a larger setting. Trial participants will complete the questionnaire at the beginning and at the end of the course. Only data from the first completion (baseline) will be used for validation purposes. Data from the second administration at the end of the course will be analysed after determining the items to be included in the validated instrument. Items remaining in the validated instrument will be used to generate baseline scores for the educational environment and to monitor and compare any possible changes at the end of the trial.

#### Development of the EBMEEM tool

Using the format of previously developed tools the content of the RHL-EBM Course were adapted and included in a draft instrument that yielded 63 Likert-scale items. The items were organized around certain themes namely, learning opportunities; own learning (EBM competence); availability of leaning resources; teachers and teaching (EBM specific); supervision and support; EBM practice (EM atmosphere); and general atmosphere. After several iterations of pilot-testing involving postgraduate trainees in a number of countries and the Steering Committee, the draft instrument to be validated and used in outcome assessement was agreed upon.

Process outcomes such as the experience of the facilitators and the postgraduate trainees will be evaluated through interviews.

### Follow-up procedure

The intervention period will be 8-12 weeks. Currently, follow-up beyond the trial period is not planned mainly due to financial constraints. If funds are available a one-year assessment of the EBM educational environment will be considered.

### Data management

Data will be collected in the centres using an online data management system developed by the Geneva Foundation for Medical Education and Research (GFMER). Data management will be based at the GFMER, Geneva, Switzerland supervised and monitored by HRP/RHR and Birmingham University.

Data collection will be at baseline (before the intervention), and after completion of the intervention. Data will be recorded by online completion of data forms (MCQs) and questionnaires (attitude, educational environment).

Data entry will be monitored on a day-to-day basis and any problems with logins, missing data and queries will be resolved promptly. The system allows data export to common statistical packages for analysis. It has been agreed that the data files will be exported to an Excel file to be analyzed by the trial statistician in the Statistics and Informatics Services Team at the HRP/RHR.

### Analysis plan

Since the random allocation is by teaching units and the inferences will be made at this level, the analysis at the teaching unit will be the unit of analysis.

At each participating unit the postgraduate trainees will use the online system to enter their personal data and access the assessment questions (all) and the e-learning modules of the course (either intervention or control materials). The postgraduate trainees will complete the relevant sections at baseline and at the end of the trial period.

### Analysis plan for primary outcomes

A score for each individual by dividing the number of points obtained in a test by the maximal number of points that can be obtained will be computed. A summary measure will be obtained for each teaching unit as the mean score for all the individuals taking the test in that unit at baseline (pre-) and post-evaluation phase.

We will compare the knowledge (main outcome) between intervention and control units using analysis of covariance, with pre-test score as a baseline covariate and terms for the stratum and for the group in the model. A 95% confidence interval will be calculated for the difference in knowledge between intervention and control, based on the error term from the analysis of covariance.

We will also compute the change in knowledge as the difference between the post- and the pre-test scores for each individual. A summary measure of the gain in knowledge for each teaching unit will be computed as the mean gain in knowledge. We will compare the gain in knowledge between intervention and control units using analysis of variance, with terms for the stratum and for the group in the model. A 95% confidence interval will be calculated for the difference in gain in knowledge between intervention and control, based on the error term from the analysis of variance.

It is anticipated that in some countries there will be existing formal or informal postgraduate training activities on EBM. These will be reported in the survey of EBM activities that will be conducted before the trial. If data permits a stratified analysis based on the presence or absence of additional basic EBM courses will be conducted. However, it is acknowledged that the study may be underpowered to provide a definitive answer according to the two strata.

### Number of subjects and statistical power

There is limited information regarding baseline EBM knowledge and possible knowledge gains and practice changes following implementation of such courses [16]. The evaluation of the pilot course developed within the Leonardo da Vinci pilot project provided some data that were used in the sample size calculation [6,7]. In the pilot evaluation only electronic modules were evaluated as a before and after analysis: For sample size calculations, the standard deviation (SD) for the average gain in knowledge (%) estimated from teaching units/modules means in different countries as SD = 7.9% (likely to be an overestimate) was taken. To allow for random variation of this estimate, a range of SD between 5% and 15% was considered. The power achieved with a total of 60 teaching units to detect a difference in gain of 10% (relative to the maximum score) between two groups in a two-sided test of 5% level, assuming SD = 10%, is 97%. If SD = 12%, the power is 90%. If SD = 14%, the power is 80%. Seven countries: Argentina (4 institutions), Brazil (20), Democratic Republic of Congo (4), India (10), Philippines (10), South Africa (6), Thailand (10) will participate in the trial whose duration is estimated in one year.

## Main problems anticipated

**Identification of facilitators**in the experimental intervention units: The principal investigator will use her/his judgement on the selection of the facilitators and provide training if appropriate. However, the whole philosophy of the Course is for the facilitators to guide the students in the clinical setting and monitor their progress in the process. The facilitators do not need to be EBM experts but rather respected clinicians that could be seen as role models or opinion leaders within their settings.

### Internet connectivity

Although all settings will have access to internet for data entry and course content (RHL-EBM) our initial contacts suggest that there may be connectivity problems in some settings like Congo DRC and South Africa. In these settings close monitoring by principal investigators will be required to ensure that emerging problems are solved rapidly.

### Existing EBM courses

It is possible that in some of the teaching units there may be other postgraduate teaching activities such as workshops or scientific conferences. It is not possible to control these activities occurring in each setting. The survey conducted before the trial will give information on the scope of the activities likely to exist in each setting. There are two possible protective mechanisms against these activities becoming a threat to the validity of trial results. First, random allocation should ensure that such activities are equally distributed in both groups. Second, stratified analysis based on the presence and absence of existing courses is planned as a secondary analysis.

## Expected outcomes of the study

If effective, the RHL-EBM Course will provide an educational intervention that can be scaled up in low and middle-income countries in both teaching and non-teaching institutions. As such it will form a core component to improve quality of care. Discussions with national and international professional organizations to implement the course with formal certification can be performed.

Improvements on knowledge gain, attitudes and skills are important for providing good quality care. Any potential impovement in educational environment will have a major impact on the quality of inservice training not only in resource-poor settings. An original EBM Educational Environment Measurement Tool will be available for all academic or nonacademic settings.

Trial results will be published in a peer-reviewed journal. The results will be disseminated to the participating centres through meetings of the research team with participants at the end of the study. The results will also be presented and disseminated through the WHO website and the WHO reproductive Health Library (RHL).

## Ethics and informed consent

The trial interventions are implemented at the institutional level. Therefore, the primary point at which permission is sought is at the institutional level. Receiving permission from the institution does not constitute a consent on behalf of the participants. It constitutes an administrative agreement between the PI and the institution. A permission sheet will be presented to the person responsible for the institution to be signed. We anticipate that in most cases this person will be the academic head of the obstetrics and gynaecology department.

The facilitators/focal persons and the postgraduate trainees will be informed that one or more educational programme(s) are being evaluated but with no further details. After random allocation each unit will be informed about the activities they should follow including completion of MCQs and other online assessments. We plan to seek consent from individuals within the participating institutions. Individuals will not be identified in the questionnaire. If *intervention 1 *is shown to be beneficial in the primary endpoints then it will be provided to all *intervention 2 *institutions.

The study protocol was ethically aproved by WHO and will also be aproved in each participatin g country.

## Competing interests

The authors declare that they have no competing interests.

## Authors' contributions

RK, KSK and AMG had the original idea for the study and drafted the first version of the research protocol. It was then discussed with all other authors in two meetings in Geneva and the correspondent inputs were added to the protocol. All have read and agreed with the final version of the current study protocol.
